# 

**Published:** 1904-09

**Authors:** 


					﻿^Twentieth J)eat.
Vol. XX.
SEPTEMBER, 1904.,
No. 3.
TEXAS
Hectical Journal.
Subscription, $i.oo a Year, in Advance.
Single Copies, io Cents.
PUBLISHED AT AUSTIN, TEXAS, BY
F. E. DANIEL, M. D.,
Proprietor.
printed by the von boeokmann-jones oo.
Entered at the Poatofflce at Austin, Texas, as Second-class Mall Matter.
				

